# Reconfigurable Integrated High-Speed Thermal Metamaterial Pixel Arrays

**DOI:** 10.1021/acs.nanolett.5c03156

**Published:** 2025-08-07

**Authors:** Yibai Zhong, Xiu Liu, Zexiao Wang, Tianyi Huang, Jingyi Zou, Sen Lin, Xiao Luo, Zhuo Li, Rui Cheng, Xu Zhang, Sheng Shen

**Affiliations:** † Department of Mechanical Engineering, 6612 Carnegie Mellon University, Pittsburgh, Pennsylvania 15213, United States; ‡ Department of Electrical and Computer Engineering, Carnegie Mellon University, Pittsburgh, Pennsylvania 15213, United States

**Keywords:** thermal signatures, metamaterial pixels, thermal emission

## Abstract

Thermal signatures carry unique infrared appearances and spectral fingerprints of objects, but controlling them across spatial, temporal, and spectral domains remains challenging due to thermal emission’s slow, diffuse, and broadband emitting nature. We demonstrate a reconfigurable ultrafast thermal metamaterial pixel array integrating active metasurfaces with dual-gate graphene transistors (Gr-FETs). Each pixel’s Gr-FETs provide heater-switch dual functionalities: one as a broadband transparent microheater supporting arbitrary metasurface designs for multicolor, narrowband infrared emission with ultrafast modulation speed of minimum 187 kHz, and the other as an electrical switch enabling unified array control without compromising emission intensity. Decoupling thermal generation and emission design processes, our approach provides unprecedented programming flexibility across space, time, and wavelength. Our fabricated array experimentally demonstrated 26 alphabetical letters through progressive scanning, paving the way for universal thermal signature control in advanced thermal-infrared applications.

## Graphical abstract

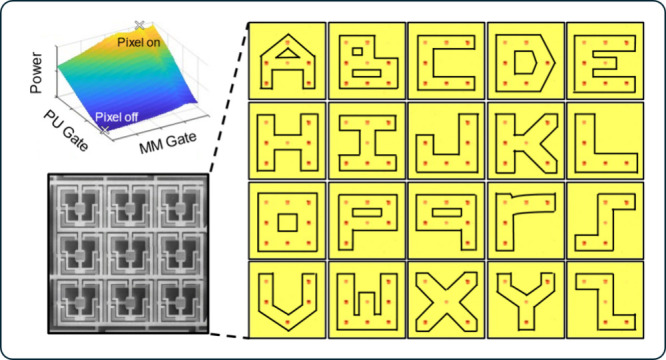

As the infrared appearance of objects, thermal signatures are fundamentally governed by thermal radiation, which carries spectral fingerprints of molecular species and spans two atmospheric transparent windows.^1–4^ The ubiquity and importance of thermal radiation underpin a broad range of applications, including active thermography and infrared therapy in medicine,^5–9^ thermal integrated photonics and active metamaterials,^10–18^ thermal camouflage, management and encryption,^19–24^ and thermal microelectro-mechanical systems (MEMS).^25–29^ However, the ability to dynamically control thermal-infrared signatures across spatial, spectral, and temporal dimensions remains elusive due to material limitations and design complexities. While various nanophotonic structures have been employed to realize spectral, directional, and polarization control of thermal emission, these implementations are generally static and limited to single-pixel demonstrations. Recently, active metasurfaces incorporating phase-change materials, electrochemical fluids, or micromechanical machines have enabled some degree of dynamic control, but they still suffer from restricted modulation contrast, slow speed, and fabrication challenges at scale. Therefore, large-area dynamic thermal pixel arrays capable of achieving high resolution spatial modulation, high contrast and fast response are highly desired for effective manipulation of thermal signatures.^30^

In the visible spectrum, the implementation of scalable pixelated arrays has been well established in visual display technologies, most notably through active-matrix electroluminescent displays utilizing thin-film transistors.^31,32^ In contrast, thermal-infrared pixel techniques remain in a nascent stage. Existing work predominantly relies on direct in-plane electrical routing, which imposes constrains on pixel density, scalability and power consumption.^33,34^ Recent advancements in passive-matrix configurations,^30,35^ wherein the pixel element is positioned at the intersection of perpendicular back column and front row electrode lines, offer partial relief by reducing wiring complexity. Yet they have been constrained by fabrication complexities and fundamentally limited by the Alt-Pleshko effect.^31,36^ The realization of active thermal-infrared pixels requires devices that combine high-speed switching, broadband infrared transparency and compatibility with emitters. Graphene field-effect transistors (Gr-FETs) are uniquely positioned to meet these demands. Owing to its atomically thin nature,^37–39^ graphene exhibits broadband transparency consistently above 90% from visible to far-infrared wavelengths, thereby minimizing its interference with the thermal radiation signals emitted or received by the integrated devices. In addition, the ultrasmall thermal mass of graphene enables ultrafast electrothermal responses.^40–43^ Nevertheless, conventional Gr-FETs suffer from substantial leakage currents due to monolayer graphene (MLG)’s semimetallic nature,^44–46^ resulting in significant emission crosstalk.^47^

In this work, we develop a reconfigurable thermal metamaterial pixel architecture that overcomes these longstanding barriers through the monolithic integration of dual gate Gr-FETs with active infrared metasurfaces. The dual-gate control of each pixel enables “heater-switch” duality for Gr-FETs, where transistors can not only behave as transparent tunable microheaters integrated with arbitrary plasmonic metamaterials for thermal emission control, but also act as analog switches for thermal power regulation, simultaneously maximizing thermal contrast and minimizing interpixel emission crosstalk. The dual-gate design enables dynamic tuning of thermal-infrared pixel power distribution and thermal emission, without the need for complex voltage routing or mechanical modulation. Our transient measurement further verifies that the switching speed of such thermal pixels can be ultrafast owing to extremely small thermal mass of MLG, without compromising its emission intensity. The time constant of the transient modulation can be as small as 1.87 μs, which can be orders of magnitude faster than previously reported devices with similar functionalities.^30,34,42,48^ Moreover, since large-area MLG can be synthesized via cost-effective low pressure chemical vapor deposition (LPCVD) production with simple carbon precursor^49–52^ and transferred onto almost all substrates for van der Waals integration,^53–55^ we experimentally realize a large-area active matrix thermal pixel array operating under a unified gate-driving scheme and demonstrate programmable emission patterns, including arbitrarily addressing each or every row of the pixels as well as full A-Z alphabet rendering through progressive scanning. By decoupling the heat control from thermal-infrared emission shaping at the pixel level, this platform introduces a scalable strategy for spatiotemporal and spectral control of infrared signals with submillisecond response, paving the way for advanced larger scale thermal displays, adaptive camouflage and high-speed infrared communication.

The cores of our system are reconfigurable thermal metamaterial pixels, designed to deliver localized, spectrally selective, and rapidly switchable infrared emission. To demonstrate the scalability and programmability of our platform, we fabricate an array of thermal metamaterial pixels as a reconfigurable infrared display. The fabricated device is wire bonded onto a chip holder (Figure 1a) and integrated into external controlling circuits. To allow for scalable integration, we arrange pixels in rows and columns with shared source-drain and gate lines, implementing the active-matrix addressing schemes used in visual display technology. As displayed in Figure 1b, the thermal metamaterial array consists of 9 pixels arranged into three rows by three columns. The parallel connection of pixels in each row realizes consistent source/drain voltage configurations across each pixel, enabling active-matrix style addressing with minimal wiring complexity. As proof of concept, we implement a progressive scanning protocol that activates specific pixels in sequence to form programmable infrared patterns. As demonstrated in Figure 1c, the 9-pixel array scans through each row of pixels with one, two or three pixels light up at the desired locations. Using this approach, we successfully render all 26 Latin alphabet letters in real-time by progressively scanning through rows of pixel combinations across the array. Each pixel consists of a central metasurface-integrated microscale graphene thermal engine (MM Gr-FET) surrounded by four peripheral-unit graphene transistors (PU Gr-FETs) configured as voltage-controlled switches. Within each pixel, the MM Gr-FET acts as the primary emitter, delivering Joule heating via source (*V*_*S*_) and drain (*V*_*D*_) to a spectrally engineered metasurface that defines the emission wavelength. The PU Gr-FETs act as tunable resistive pathways that gate power delivery to the MM device, enabling a high degree of thermal contrast through selective voltage gating. The bottom gates and the source/drain lines for all transistors are fabricated of Au with aluminum oxide (Al_2_O_3_) as the dielectric layer and thermal oxide (SiO_2_) Si wafer as the substrate. All the fabrication steps are compatible with the complementary metal-oxide-semiconductor (CMOS) process, as discussed in Supporting Information 5.

**Figure 1:**
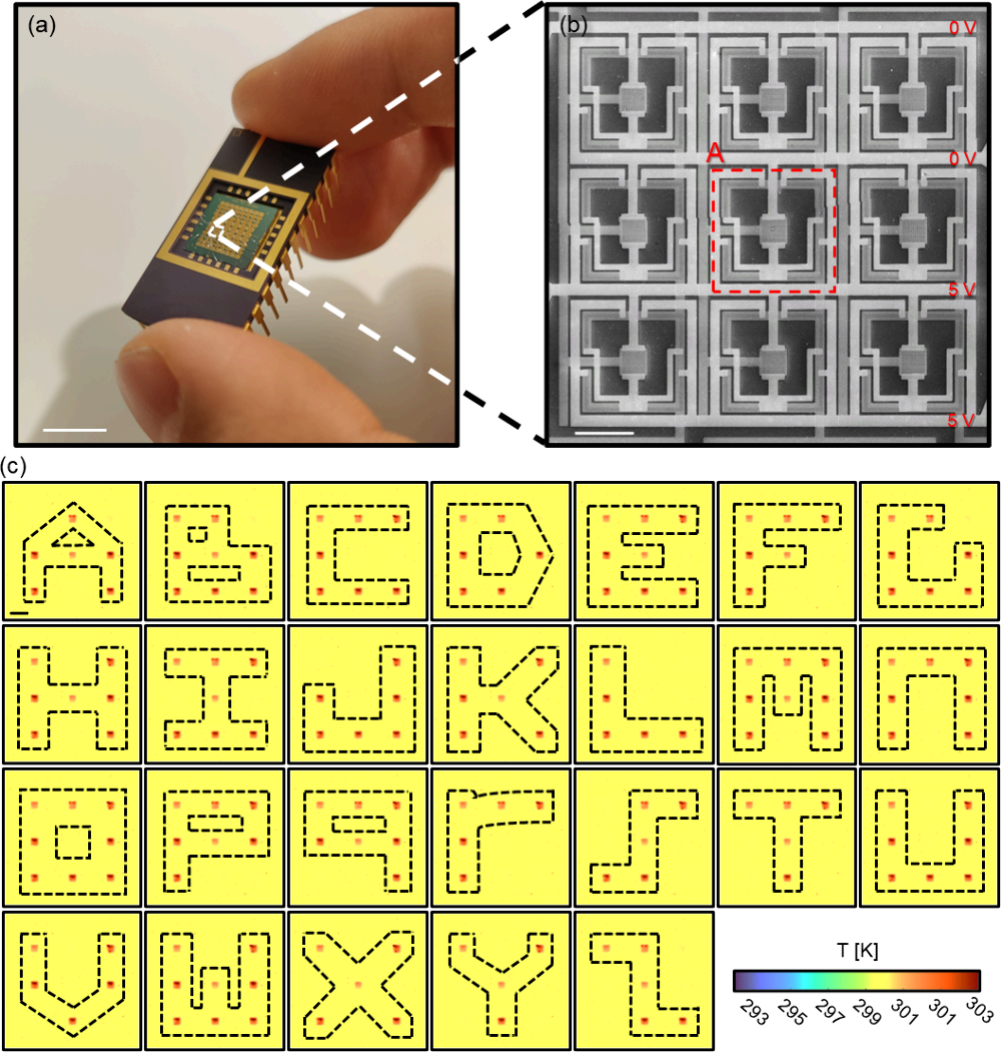
Reconfigurable three-by-three thermal metamaterial pixel array. (a) Wire-bonded devices onto a 24-pin chip holder containing multiple three-by-three thermal metamaterial pixel arrays (scale bar: 90 mm). (b) Zoomed-in scanning electron microscopy (SEM) image of a three-by-three thermal metamaterial pixel array (scale bar: 50 μm), composed of pixel rows with parallel connectivity and shared control gates along the column, enabling a unified gate-driving scheme. Each thermal metamaterial pixel unit consists of an MM Gr-FET located in the center of the pixel and four L-shaped PU Gr-FETs surrounding it. Four analog voltage inputs are needed to operate one pixel: the four PU Gr-FETs are connected in parallel with each other and are controlled by the same gate (*V_G,PU_*). The MM Gr-FET is controlled by another gate (*V*_*G,MM*_) and connects in series with the PU Gr-FETs. Each pixel on the same row shares the same *V*_*S*_ and *V*_*D*_. (c) Progressive scan across the array rendering letters A-Z (scale bar: 50 μm). The 5 V potential difference created between one selected pair of source/drain lines enables individual pixel or full-row addressing with tuning of gate voltages.

To validate the ability of individually turning on and off individual pixel units, a single thermal metamaterial pixel is fabricated and characterized. As demonstrated in the false-color SEM image (Figure 2a), the four PU transistors parallelly connect the *V*_*D*_ of the pixel on one side and the MM transistor on the other side, which then connects the *V*_*S*_ of the pixel via the MM transistor. Since *V*_*D*_ > *V*_*S*_ is commonly set for the pixel, the four PU Gr-FETs can effectively pull up the potential across the MM transistor when their channel resistances are lowered by the shared bottom gates (*V*_*G,PU*_). Meanwhile, the MM transistor controlled by another bottom gate *V*_*G,MM*_ can have their channel resistance varied simultaneously to increase or decrease their surface temperature rise. Therefore, when the pixels are assembled into arrays, instead of allocating individual gates for each pixel, the pixels along the same *y*-direction share the same gate lines, therefore significantly reduces the control complexity. Yet each pixel remains individually addressable as long as power inputs are applied across only one designated row in progressive scanning, crossing the two gate voltage potentials applied to the desired pixel column.

**Figure 2:**
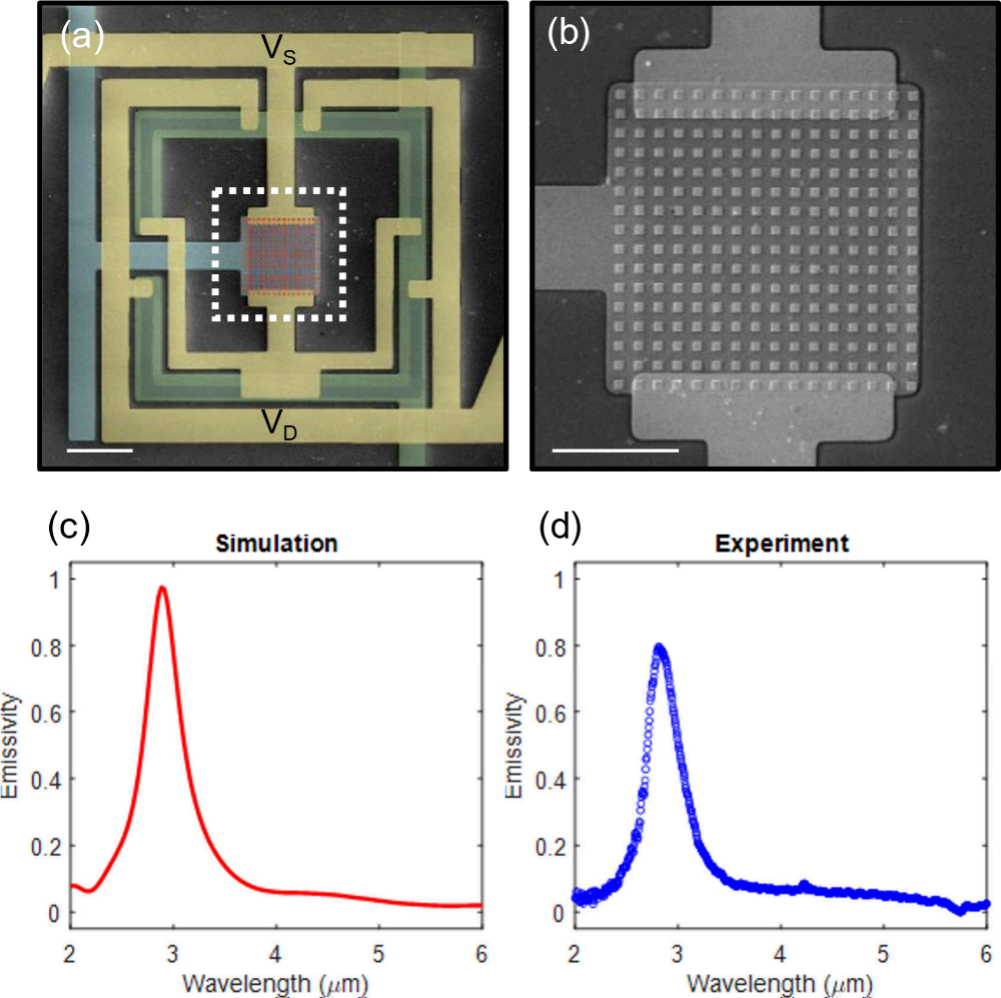
Reconfigurable ultrafast thermal metamaterial pixel unit. (a) SEM image with false colors of a thermal metamaterial pixel unit (scale bar: 20 μm). The source/drain lines are colored yellow, and the graphene area are colored blue. The cyan and green colors represent gate electrodes for the MM Gr-FET and PU Gr-FETs. (b) Zoomed-in view showing the metamaterial on top of the MM Gr-FET (scale bar: 10 μm). (c) Emissivity simulation of Au metasurfaces with side length of 500 nm. (d) The experimental measurement of a fabricated metasurface with side length of 500 nm shows good agreement with the simulation.

Since thermal emission results from the product of emissivity and Planck blackbody radiation, we strategically design the pixel region (Figure 2b) with metamaterials exhibiting near-unit emissivity resonance, while the surrounding PU regions remain spectrally neutral due to graphene’s low emissivity and infrared transparency. This configuration enhances emission contrast, complementing the temperature difference achieved through our pixel circuit architecture. The metamaterials are also highly customizable – the geometry variation of their units would lead to narrow-band thermal emission peaking at designated mid-infrared wavelength of 2.9 μm, as demonstrated and validated in Figures 2c and 2d. By separating the mechanisms of heat generation and spectral emission, this pixel architecture enables unmatched flexibility in dynamically programming thermal output across spatial, temporal, and spectral regimes.

To further evaluate the electrothermal performance of the pixel array, we characterize the electrical and material properties of the monolayer graphene synthesized via LPCVD (details in Supporting Information 5). Gr-FET test structures, as shown in Figure 3a-inset, are fabricated following the same fabrication steps as the pixelated devices (details in Supporting Information 5) to assess their carrier mobility and contact resistance. The channel width is 20 μm with four different channel lengths of 5 μm, 10 μm, 20 and 50 μm so that the contact resistance and mobility of MLG can be measured via transmission line method (TLM).^56^ When a constant source-drain voltage *V*_*DS*_ = 50 mV is applied across each of the channels, the contact resistance of the device is estimated to be around 200 Ω (detailed in Supplementary Note 1), which is more than 1 order of magnitude smaller than that of the graphene film and hence can be neglected. The field-effect carrier mobility of graphene μ can be modeled using the Drude model with the *I*_*DS*_-*V*_*G*_ measurement results shown in Figure 3a:

1 $$\mu =\frac{L}{W}\frac{1}{C_{OX}^{\prime }}\frac{1}{V_{DS}}\frac{dI_{DS}}{dV_{G}}$$

where *L* and *W* are channel length and width of the Gr-FET, *I*_*DS*_ and *I*_*G*_ are the measured channel current and gate voltage set for the Gr-FET, and *C*_*OX*_^′^ = *ε*_*r*_ *ε*_0_/*d* representing the capacitance of Gr-FET per unit area with *ε*_*r*_ equaling the relative permittivity of Al_2_O_3_ dielectric layer and *ε*_0_ being the vacuum permittivity. The maximum hole mobility is estimated to be around 1100 cm^2^/(V s), with the Dirac point being close to neutral gate voltage, which indicates a good carrier mobility with minimum doping for LPCVD-synthesized graphene. Raman spectroscopy confirms monolayer graphene’s quality and low defect density (Supplementary Note 2), validating that the LPCVD graphene is suitable for high performance thermal modulation.

**Figure 3:**
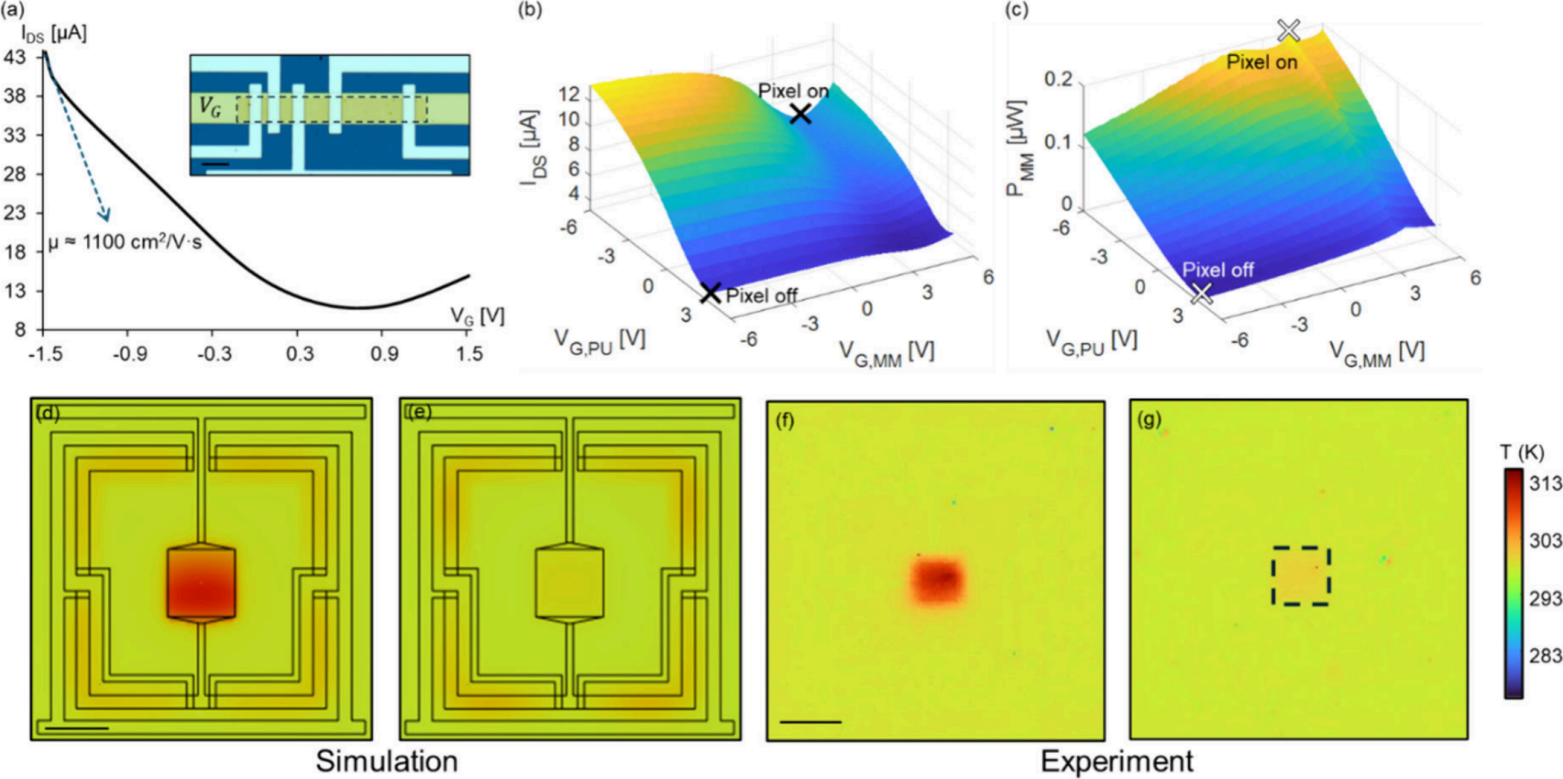
Characterization of the MLG and single thermal metamaterial pixels. (a) Electrical *I*_*DS*_-*V*_*G*_ characterization with *V*_*DS*_ = 50 mV on graphene FET test structures (inset with scale bar: 20 μm). The maximum charge carrier mobility estimated from the curve gradient is 1100 cm^2^/(V s). (b) Dual-gate electrical characterization of the thermal metamaterial pixel. The *I*_*DS*_-*V*_*G*_ measurement shows that the overall channel resistance of the pixel increases as V_G,PU_ changes from −6 to 3 V. The variation of *V*_*G,MM*_ further changes the distribution of internal power, turning the pixel off by decreasing the channel resistance of the heater transistor when the overall channel resistance is high (“Pixel off”), and vice versa (“Pixel on”). (c) Calculated power distribution on the MM Gr-FET verifies the aforementioned configurations. (d) to (g) Steady-state simulation and experiment thermal characterization of the pixel (scale bar: 20 μm). *V*_*DS*_ = 7 V. *V*_*G,MM*_ = 7.9 V, *V*_*G,PU*_ = −4.4 V when pixel-on, *V*_*G,MM*_ = −8.6 V, *V*_*G,PU*_ = 4.2 V when pixel-off.

Based on high quality MLG, the geometric design of the fabricated thermal metamaterial pixels shown in Figure 2a is further optimized and finalized. Each pixel shall consist of a central square-shaped MM Gr-FET with a side length of 20 μm, covered by Au metasurface layer serving as an emitter. Surrounding the MM Gr-FET are four L-shaped PU Gr-FETs, left optically transparent to minimize background emission. The four PU Gr-FETs, when connected in parallel, are designed with a larger surface area than the MM transistor to dissipate residual heat. Under identical gate voltage, the PU Gr-FETs exhibit nearly four times higher resistance than the MM Gr-FET. The electrical conductivities of the two sets of Gr-FETs (*σ*_*MM*_ and *σ*_*PU*_) are governed by both field-induced charge carriers (tunable via gate bias) and residual carriers (*n*_*res*_) accounting for charge transport near the Dirac point.^44,46^ These conductivities can be modeled as

2 $$\{\begin{array}{l} \sigma_{MM}=n_{res}e\mu +C_{ox}^{\prime }\left|V_{G,MM}-V_{Dirac,MM}\right|\mu \\ \sigma_{PU}=n_{res}e\mu +C_{ox}^{\prime }\left|V_{G,PU}-V_{Dirac,PU}\right|\mu \end{array}$$

where *V*_*G*_,_*MM*_-*V*_*Dirac*_ _,*MM*_ and *V*_*G,PU*_-*V*_*Dirac*_ _,*PU*_ are the potential difference between gate voltages and Dirac points of MM Gr-FET and PU Gr-FET, respectively. The *n*_*res*_ *eμ* term captures the residual conductivity of graphene at the Dirac point, which arises from diffusive transport driven by carrier density fluctuations, induced by charged impurities typically located in the substrate or at the graphene-substrate interface.^57^ Assuming negligible contact resistance, the total channel resistance of the thermal metamaterial pixel can hence be expressed as

3 $$R_{channel}=\frac{1}{\sigma_{MM}}\frac{L_{MM}}{W_{MM}}+\frac{1}{\sigma_{PU}}\frac{L_{PU}}{W_{PU}}$$

where $\frac{1}{\sigma_{MM}}\frac{L_{MM}}{W_{MM}}=R_{MM}$ , $\frac{1}{\sigma_{PU}}\frac{L_{PU}}{W_{PU}}=R_{PU}$ , accounting for the contributions from the MM Gr-FET and the PU Gr-FETs, respectively. The effective geometric aspect ratio $\frac{L_{MM}}{W_{MM}}=1$ and $\frac{L_{PU}}{W_{PU}}=3.91$ . Therefore, the thermal metamaterial pixel can be turned on and off via concurrent gate tuning of both the MM Gr-FET (*V*_*G,MM*_) and the PU Gr-FETs (*V*_*G,PU*_): as measured in Figure 3b, when *V*_*DS*_ = 50 mV and *V*_*G,PU*_ = 3 V (set close to the Dirac point of the PU Gr-FETs), the overall *I*_*DS*_ of the pixel is small regardless of *V*_*G,MM*_ due to the dominant larger resistance of PU Gr-FETs. Further biasing *V*_*G,MM*_ negatively reduces the MM Gr-FET channel resistance, resulting in the majority of the voltage drop to occur across the PU Gr-FETs, minimizing the power dissipation in the MM Gr-FET microheater and effectively turning the pixel off, as evidenced by the measured *I*_*DS*_ in Figure 3b using eqs 2 and 3 and demonstrated in Figure 3c (detailed calculation in Supplementary Note 3). On the contrary, when *V*_*G,MM*_ = 4 V (near the Dirac point of the MM Gr-FET) and when *V*_*G,PU*_ is set negatively away from the Dirac point, the pixel can be turned on and the power is funneled into the MM Gr-FET microheater. This configuration results in an increase in the total *I*_*DS*_ compared with the off case (Figure 3b), and localizes heating at the emitter, as shown in Figure 3c.

To further model and optimize the pixel’s thermal response under dual-gate control, we establish a steady state thermoelectrical simulation via COMSOL Multiphysics. The source-drain voltage *V*_*DS*_ is set to a constant 7 V, while the background temperature is set to 298 K. The model reveals strong thermal localization in the MM Gr-FET under appropriate gate biasing. The pixel is turned on when *V*_*G,MM*_ = 7.9 V and *V*_*G,PU*_ = −4.4 V, as shown in Figure 3d, where the MM Gr-FET reaches a temperature rise of nearly 15 K while the PU Gr-FETs have a lower temperature rise of 3 K. On the other hand, when *V*_*G,MM*_ = −8.6 V and *V*_*G,PU*_ = 4.2 V, the pixel is turned off as displayed in Figure 3e, where temperature rise at the MM Gr-FET is 2 K while the PU Gr-FETs maintain a temperature rise of around 3 K. Such predicted thermal performance is further verified by thermal mapping under an infrared camera shown in Figures 3f and 3g. Notably, the residual heat from the PU Gr-FETs is consistently invisible under thermal mapping due to the high transparency of monolayer graphene and the low emissivity of Au underneath. Meanwhile, >15 K temperature rise from the MM Gr-FET is captured when the pixel is on, while its temperature rise is <3 K when the pixel is turned off, demonstrating high spatial thermal contrast controlled purely by electrostatic gating. The lack of visible emission from the PU Gr-FETs further confirms the high IR transparency of monolayer graphene and the spectral selectivity of the metasurface design. This new methodology paves the way for implementing large scale infrared pixel arrays without compromising control capability or thermal contrast. To assess the switching speed and cutoff frequencies of the thermal metamaterial pixels, time-domain thermoreflectance measurements are conducted with respect to the MM transistor. As plotted in Figure 4a, by alternating the gate voltages between the on-state pair (*V*_*G,MM*_ = 3 V and *V*_*G,PU*_ = −6 V) and off-state pair (*V*_*G,MM*_ = −9 V and *V*_*G,PU*_ = 3 V) every 7.5 μs, with *V*_*DS*_ is maintained at 5 V, we measure the temporal evolution of pixel heating and cooling. The simulated temperature rise and the measured reflectance change exhibit excellent agreement in their dynamics. When turned on and off, transient simulations show the 10–90% rising time *t*_*r*_ and falling time *t*_*f*_ of 2.73 and 2.77 μs, respectively, corresponding to a minimum 3-dB cutoff frequency of *f*_*c*_ = 0.35/*t*_*f*_ = 126 kHz.^47^ As captured in Figures 4b to 4e (image capturing detailed in Supporting Information 5), the reflectance change Δ*R*/*R* captured from the heater surface is found to decrease when the pixel switches from off to on state and increase back to zero when the pixel turns off (Supplementary Video), with the 10–90% rising time being 1.87 μs and the falling time being 1.33 μs, corresponding to a minimum 3-dB cutoff frequency of 187 kHz. These results confirm that the pixel switching is governed by the ultralow thermal mass of monolayer graphene, which is a key advantage compared with the slower time scales typical of electrochemical materials or bulk heaters.^58–61^

**Figure 4:**
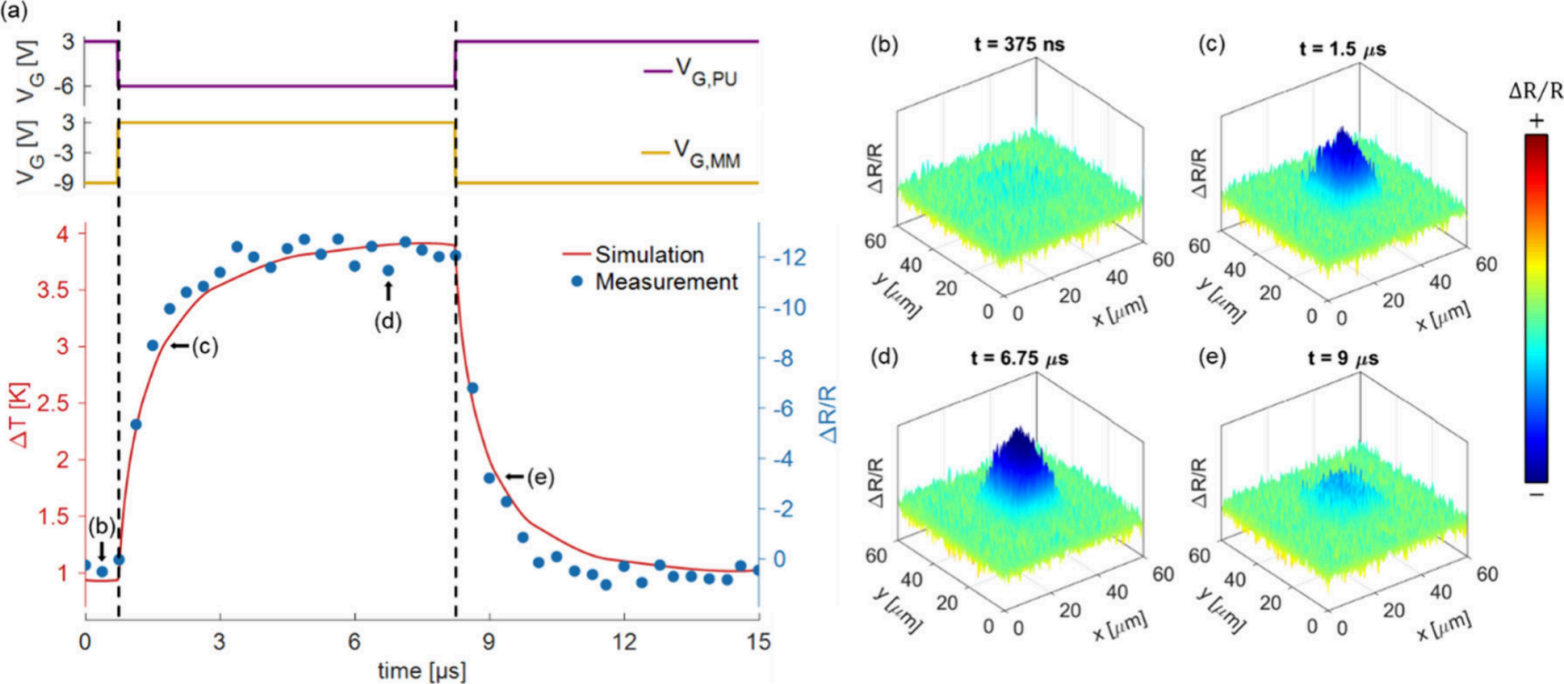
Transient analysis of single thermal metamaterial pixels. (a) Temperature simulation and thermoreflectance measurement of the MM Gr-FET area under square-wave gate voltage (*V*_*G*__,*PU*_ and *V*_*G*__,*MM*_) modulations (15 μs period; 50% duty cycle), with key frames of interest captured and plotted in (b) to (e). *V*_*DS*_ is maintained at constant 5 V for both analyses. The minimum 3-dB cutoff frequencies are calculated to be 126 kHz for the simulation and 187 kHz under measurement.

With the fast transient response of pixels combined with flexible control schemes, high-speed progressive scanning for the entire thermal metamaterial pixel array to form dynamic patterns hence becomes viable. Such array configuration also allows pixel-level switching without setting a series of different source-drain bias for pixels located differently, dramatically reducing circuit complexity and power overhead as arrays scale. Take the center pixel A shown in the dashed box of Figure 1b as an example, to turn it on and keep the remaining pixels off with maximum power line potential of 5 V being set for scanning operation, the middle row where pixel A is located can have a constant *V*_*DS*_ = *V*_*D*_ – *V*_*S*_ = 5 V (*V*_*D*_ = 5 V and *V*_*S*_ = 0 V). For the row above pixel A, all the source/drain lines share the same voltage as *V*_*S*_, and for the row below pixel A, *V*_*D*_ is set for the remaining source/drain lines. Therefore, no other pixel rows are illuminated because of the net zero voltage drop. *V*_*G,MM*_ = 3 V and *V*_*G,PU*_ = −6 V are hence applied to the gate columns for pixel A to turn it on, while *V*_*G,MM*_ = −9 V and *V*_*G,PU*_ = 6 V are maintained for the other columns to mute the remaining pixels on the same row. Similar procedures can be followed for any other pixels, which are demonstrated in Supplementary Note 4. Moreover, one can also set *V*_*G,MM*_ = 3 V and *V*_*G,PU*_ = −6 V for multiple gate voltage columns, effectively turning on many or even all the pixels on the same row simultaneously without compromising their intensities (details shown in Supplementary Note 4), where the progressive scanning scheme realizing dynamic thermal display becomes viable. Such versatile operation successfully demonstrates the feasibility of using our architecture for reconfigurable thermal displays and dynamic thermal encoding at the microscale.

In summary, we demonstrate a CMOS-compatible platform for reconfigurable pixelated thermal metamaterial arrays that achieve ultrafast, high contrast and spectrally selective infrared emission via electrostatic gating of dual-function Gr-FETs. With three-by-three thermal metamaterial pixel arrays, we demonstrate the display of all the 26 Latin alphabet letters via progressive scanning. The ultralow emissivity of MLG decouples temperature and emission patterns and enables flexible integration of various thermally excited infrared emitting materials such as metasurfaces and nanoparticles with diverse materials and geometries, thus allowing highly customizable spectrum, polarization, and direction of the infrared emission. Our demonstration of submillisecond switching, narrowband spectral tunability, and programmable 2D pattern formation establishes a new class of active thermal devices with unique capabilities in space, time, and frequency domains, thus addressing the increasing demand of advanced infrared and thermal applications at micro and nanoscale.

## Supplementary Material





## Notes

Contact resistance measurement, Raman spectroscopy of graphene, calculation of electrical conductivity and channel resistance of thermal metamaterial pixels, thermal mapping results, and materials and methods (PDF)
Thermoreflectance measurement (MP4)
