# Supplementary material for: Reconfigurable Integrated High-Speed Thermal Metamaterial Pixel Arrays
Source: Nano Lett. 2025 Aug 7;25(33):12712–8. doi: 10.1021/acs.nanolett.5c03156 (PMC12371864; doi:10.1021/acs.nanolett.5c03156)
Supplement: Supplementary file 1 [file nl5c03156_si_001.pdf]

Supplementary Information of

Reconfigurable Integrated High-speed Thermal Metamaterial Pixel Arrays

Yibai Zhong[1], Xiu Liu[1], Zexiao Wang[1], Tianyi Huang[1], Jingyi Zou[2], Sen Lin[2], Xiao Luo[1], Zhuo Li[1], Rui Cheng[1], Xu Zhang[1,2,], Sheng Shen[1,*]*

[1]Department of Mechanical Engineering, Carnegie Mellon University, Pittsburgh, PA, USA

[2]Department of Electrical and Computer Engineering, Carnegie Mellon University, Pittsburgh, PA, USA

Supplementary Information 1: Contact resistance measurement

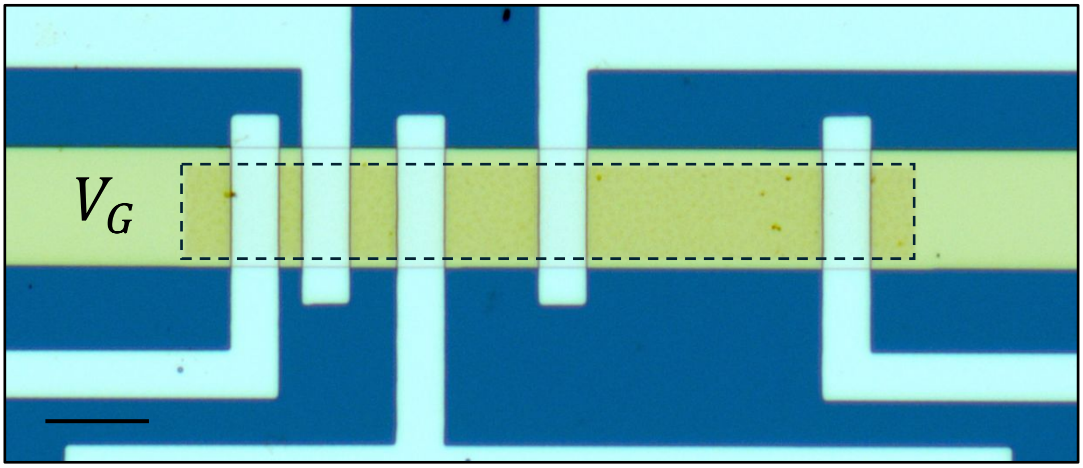

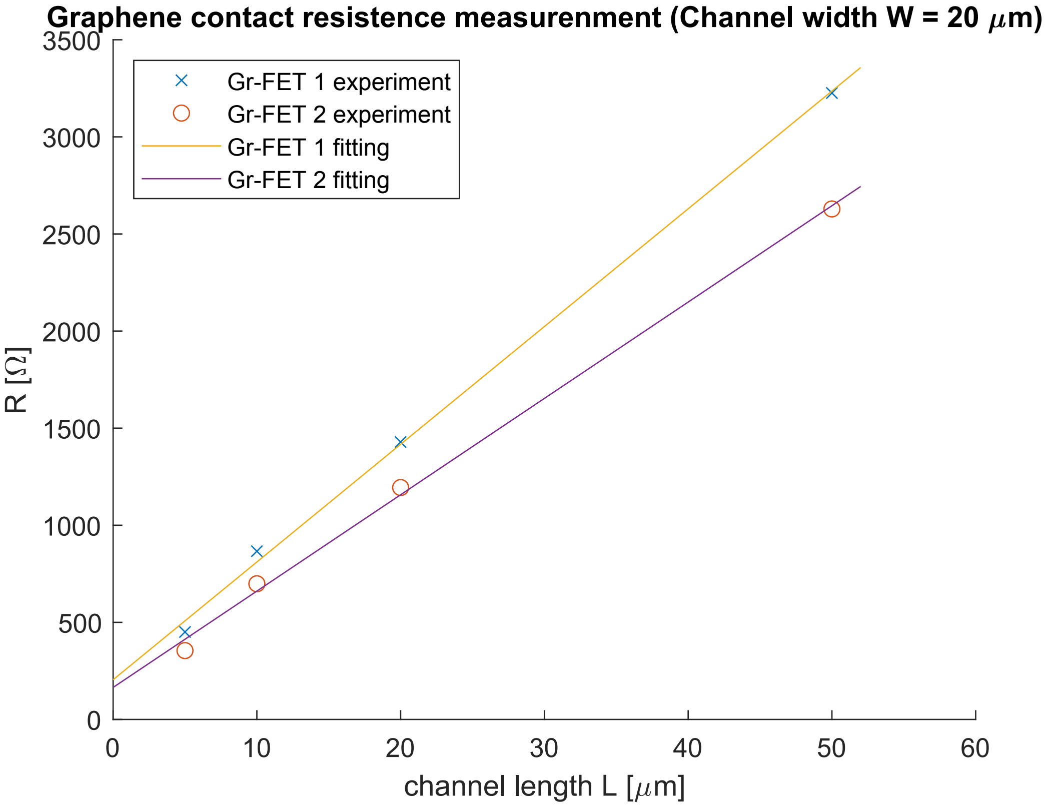

The contact resistance measurement is conducted on the Gr-FET test structure as shown in Fig. 2a-i (above). V_G is kept grounded throughout the measurement, while V_{DS} is kept as 50 mV between each pair of electrodes with channel length of 5 µm, 10 µm, 20 µm and 50 µm. Two ribbon Gr-FETs are measured, and the fitted linear lines indicate contact resistance of 164 Ω and 204 Ω respectively.

Supplementary Information 2: Raman spectroscopy of graphene

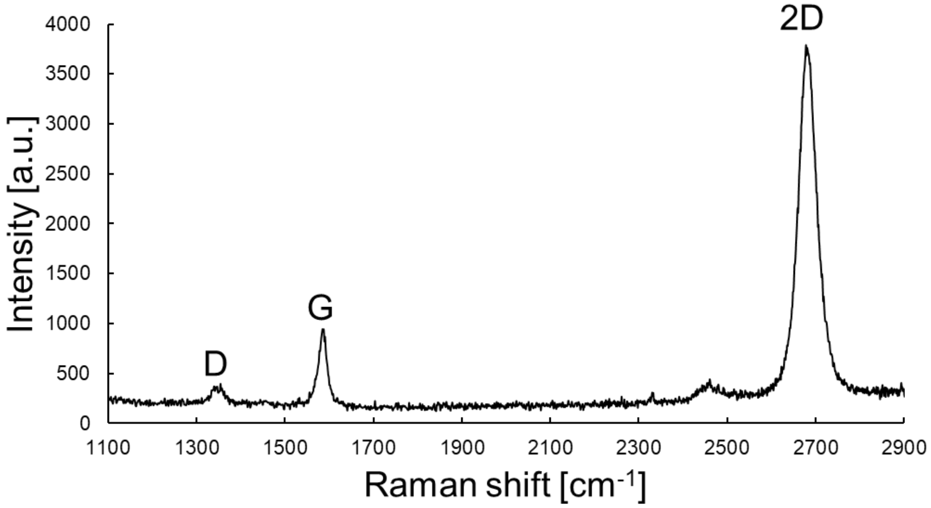

Raman spectroscopy is conducted using a 532 nm laser, where the intensity of the 2D peak around 2680cm^{-1} is more than twice compared with the G peak around 1585cm^{-1}, while the D peak at 1355cm^{-1} has a much lower intensity. Such spectrum confirms monolayer graphene existence with low defect and doping.

Supplementary Information 3: Calculation of electrical conductivity and channel resistance of thermal metamaterial pixel

As semimetal, the Drude model of conductivity for graphene describes:

$$\sigma = (n_{res}e + n_{field}e)(1)$$

, where n_{res} and n_{field} represent the charge carrier density introduced from the two major sources: residual charges as well as field-induced charges, e is the elementary charge and μ is the charge carrier mobility. The $n_{res}e\,\mu$ term essentially represents the residual conductivity of graphene at the Dirac point, arising from diffusive transport driven by carrier density fluctuations. These charge fluctuations in graphene are induced by charged impurities, typically located in the substrate or at the graphene-substrate interface. Here we assume that electron and hole share the same carrier mobility in graphene. Since Gr-FETs can be treated as parallel-plate capacitors:

$$n_{field}e = C'_{ox}V_{gate}(2)$$

, where $C'_{ox} = \frac{\varepsilon_0\varepsilon_r}{d}$ represents the oxide capacitance per unit area, and V_{gate} is the local gate voltage for graphene with respect to the Diract point. Eqns. 1 and 2 give conductivity for both Gr-FETs:

$$\begin{cases} \sigma_{MM} = n_{res}e\mu + C'_{ox}|V_{G,MM} - V_{Dirac,MM}|\mu \\ \sigma_{PU} = n_{res}e\mu + C'_{ox}|V_{G,PU} - V_{Dirac,PU}|\mu \end{cases}(3)$$

, where $V_{G,MM} - V_{Dirac,MM}$ and $V_{G,PU} - V_{Dirac,PU}$ are the potential difference between gate voltages and Dirac points of MM Gr-FET and PU Gr-FET respectively. Hence when the length and width of both transistors have their aspect ratio as $\frac{L_{MM}}{W_{MM}}$ and $\frac{L_{PU}}{W_{PU}}$, the total channel resistance of one pixel neglecting the contact resistance follows:

$$R_{channel} = \frac{1}{\sigma_{MM}}\frac{L_{MM}}{W_{MM}} + \frac{1}{\sigma_{PU}}\frac{L_{PU}}{W_{PU}}(4)$$

When both $V_{G,MM} = V_{Dirac,MM}$ and $V_{G,PU} = V_{Dirac,PU}$, $\sigma_{MM} = \sigma_{PU} = n_{res}e\mu$. Therefore, from the electrical measurement in Fig. 2b:

$$n_{res}e = \left(\frac{L_{MM}}{W_{MM}} + \frac{L_{PU}}{W_{PU}}\right)\frac{1}{R_{channel}\mu} \cong 0.003\ \mathrm{C/m^2}(5)$$

, with $\frac{L_{MM}}{W_{MM}} = 1$, $\frac{L_{PU}}{W_{PU}} = 3.91$, $\mu \cong 1100\mathrm{cm^2/V{\cdot}s}$. Meanwhile since the electrical measurement in Fig. 2b gives the total resistance $R_{channel} = \frac{V_{DS}}{I_{DS}}$ with $V_{DS} = 50$ mV, the channel resistance for individual Gr-FETs can be calculated via Eqns. 3 and 4:

$$\begin{cases} R_{MM} = R_{channel} \dfrac{\dfrac{L_{MM}}{W_{MM}}\left(C'_{ox}|V_{G,PU} - V_{Dirac,PU}| + n_{res}e\right)}{\dfrac{L_{MM}}{W_{MM}}\left(C'_{ox}|V_{G,PU} - V_{Dirac,PU}| + n_{res}e\right) + \dfrac{L_{PU}}{W_{PU}}\left(C'_{ox}|V_{G,MM} - V_{Dirac,MM}| + n_{res}e\right)} \\ R_{PU} = R_{channel} \dfrac{\dfrac{L_{PU}}{W_{PU}}\left(C'_{ox}|V_{G,MM} - V_{Dirac,MM}| + n_{res}e\right)}{\dfrac{L_{MM}}{W_{MM}}\left(C'_{ox}|V_{G,PU} - V_{Dirac,PU}| + n_{res}e\right) + \dfrac{L_{PU}}{W_{PU}}\left(C'_{ox}|V_{G,MM} - V_{Dirac,MM}| + n_{res}e\right)} \end{cases} \quad (6)$$

Where R_{MM} and R_{PU} represents the channel resistance of MM Gr-FET and PU Gr-FET respectively. $C'_{ox} = \frac{\varepsilon_r \varepsilon_0}{d}$, with $\varepsilon_r = 7.5$ for Al_2O_3 and $d = 40$ nm.

The power of MM Gr-FET can hence be estimated by $P_{MM} = R_{MM} \times I_{DS}^2$, where I_{DS} is obtained from the measurement results shown in Fig. 2b. The distribution of P_{MM} is hence demonstrated in Fig. 2c where each date point is calculated using measured I_{DS} under different $V_{G,MM}$ and $V_{G,PU}$ inputs.

Supplementary Information 4: Thermal mapping results

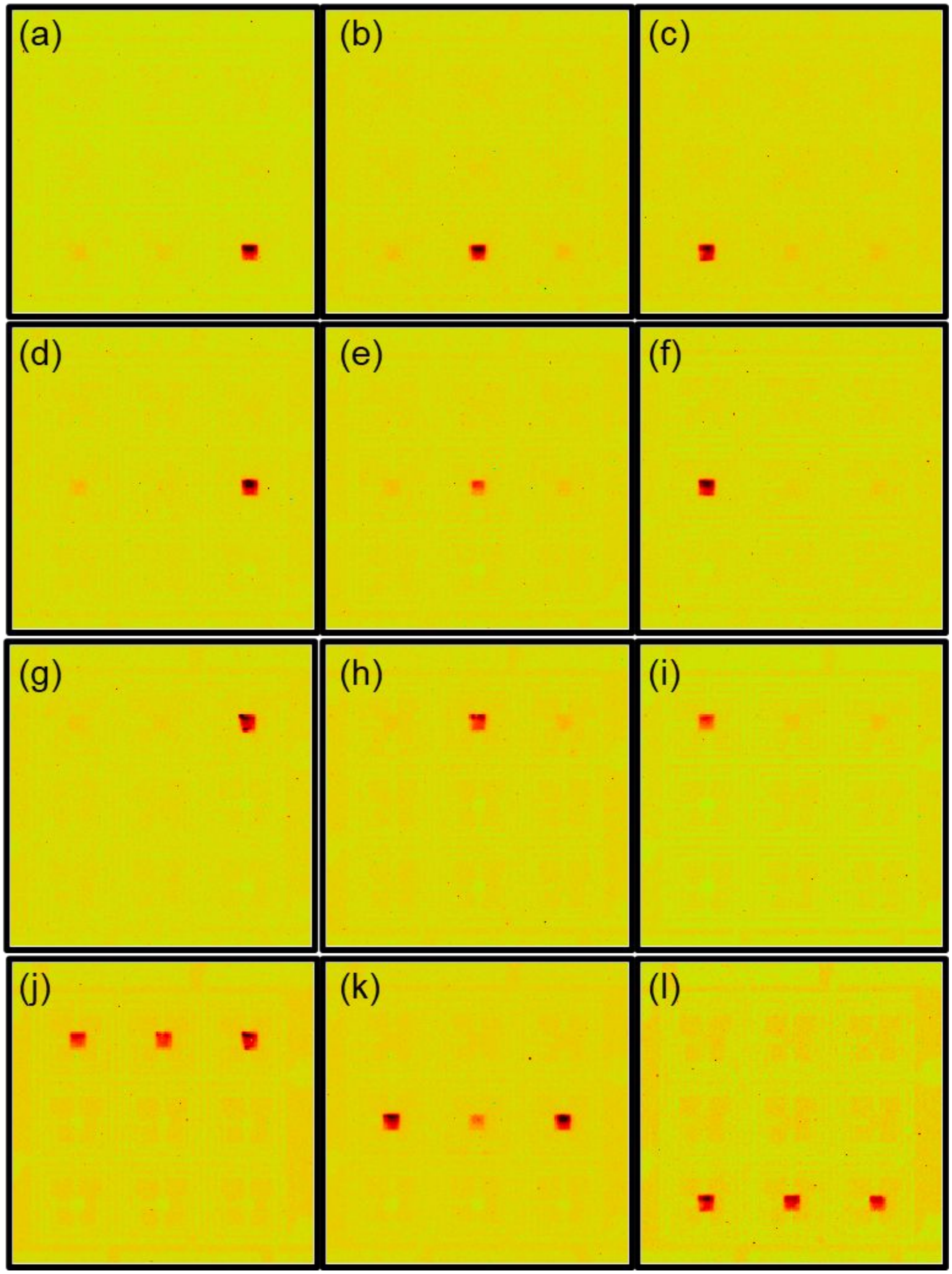

Thermal mapping results show the operation of the thermal metamaterial pixel array: (a) to (i) show the single-pixel mode where one of the nine pixel is turned on and illuminate, and (j) to (l) demonstrate the progressive scanning mode where one row of the array is turned on without compromising their emission intensity.

Supplementary Information 5: Materials and Methods

Graphene Synthesis

The MLG is synthesized via LPCVD using a 2-inch-diameter quartz tube furnace. The substrate is 99.9% pure Cu foil (25 μm thick) which is cut into one-inch-square pieces and polished via electrochemical polishing for 2.5 hours in 85% phosphoric acid as electrolyte. Ultra-high purity hydrogen with flow rate of 70 standard cubic centimeters per minute (sccm) is purged into the LPCVD chamber when the furnace is heated up to 1000 °C in 15 minutes. The temperature and hydrogen flow are maintained for another 30 minutes to further anneal the Cu foil. Then the ultra-high purity methane gas acting as the carbon precursor is mixed into the chamber with hydrogen: methane = 65 sccm: 5 sccm to maintain the total flow rate for another 30 minutes. Methane molecules hence decompose into a mixture of hydrogen, hydrocarbon and carbon with Cu as catalyst, leaving MLG deposited onto the Cu substrate. After the MLG deposition is finished, the methane flow is turned off and the furnace is opened to allow a rapid cooling process, with a flow of hydrogen of 200 sccm until room temperature is reached.

Device Fabrication

The Gr-FET is fabricated on a Si wafer with 1 μm thick thermal oxide. Photolithography followed by e-beam evaporation of 50 nm thick Au is used to fabricate the bottom gate electrode with metal lift-off. Thermal atomic-layer deposition (ALD) is used to deposit an Al_2O_3 dielectric layer of 40 nm thickness. The LPCVD-synthesized MLG is wet transferred to the top of the dielectric layer with PMMA as supporting layer and patterned to desired shape via the same photolithography process and reactive-ion etching (RIE). Lastly, similar metal deposition procedures are followed to form the source and drain electrodes. E-beam lithography is used for defining Au metasurfaces to enhance infrared emission, followed by e-beam evaporation (50 nm Au) and metal lift-off processes.

Thermal Mapping and Thermoreflectance Measurements

A QFI InfraScope is employed to capture dynamic thermal mapping images. The thermal pixels are coated with a layer of Au metasurfaces exhibiting an emissivity close to unity at wavelength of 2.9 μm, ensuring compatibility with the 2.0-4.5 μm response range of the thermal mapping system. The thermoreflectance measurement employs a 530-nm laser, where Au emitter is witnessed to have the largest negative reflectance-temperature gradient under such incident wavelength[1–3]. Two arbitrary-wave generators are synchronized by a master-slave trigger cable in finite-burst mode to simultaneously modulate the two gate

voltages following a square wave (15 μs period; 50% duty cycle). The movie mode is used to record and extract all captured frames during the modulation process.

REFERENCES

1. Ashley, E. J. & Bennett, J. M. Infrared Reflectance and Emittance of Silver and Gold Evaporated in Ultrahigh Vacuum. *Applied Optics, Vol. 4, Issue 2, pp. 221-224* 4, 221–224 (1965).

2. Cardenas, C., Fabris, D., Tokairin, S., Madriz, F. & Yang, C. Y. Thermoreflectance measurement of temperature and thermal resistance of thin film gold. *J Heat Transfer* 134, (2012).

3. Burzo, M. G., Komarov, P. L. & Raad, P. E. Optimized thermo-reflectance system for measuring the thermal properties of thin-films and their interfaces. *Annual IEEE Semiconductor Thermal Measurement and Management Symposium* 2006, 87–94 (2006).

Supplementary Video: Thermoreflectance measurement

Movie mode showing all the captured frames of the thermal metamaterial pixel thermoreflectance measurement ($\Delta R/R$) under a square-wave gate voltage modulation (15 μs period; 50% duty cycle). The pixel is turned on at 750 ns and turned off at 8.25 μs. The blue color representing a decrease of $\Delta R/R$ indicates an increase in temperature, and vice versa for the red color.
